# Multi-insecticide susceptibility evaluation of dengue vectors *Stegomyia albopicta* and *St. aegypti* in Assam, India

**DOI:** 10.1186/s13071-015-0754-0

**Published:** 2015-03-03

**Authors:** Kavita Yadav, Bipul Rabha, Sunil Dhiman, Vijay Veer

**Affiliations:** Defence Research Laboratory, Tezpur, Assam 784 001 India

**Keywords:** *St. albopicta*, *St. aegypti*, Dengue vector, Northeast India, Insecticide resistance

## Abstract

**Background:**

Dengue is rapidly expanding mosquito-borne viral infection globally facing operational challenges due to insecticide resistance in dengue vectors. We have studied the susceptibility status of potential dengue vectors *St. albopicta* and *St. aegypti* to the commonly used insecticides.

**Methods:**

*Stegomyia* larval bioassays were carried out to determine LC_10_, LC_50_ and LC_99_ values and resistance ratios (RR_50_ and RR_99_) for temephos. Adult susceptibility bioassay to 4% DDT, 0.05% deltamethrin, 5% malathion was assessed following standard procedure to determine the corrected mortality. Knock-down times (KDT_50_ and KDT_99_) were estimated and the knock-down resistance ratios (KRR_50_ and KRR_99_) were calculated.

**Results:**

*St. albopicta* wild population (WP) of Sotia was resistant to temephos as the LC_99_ value was 0.12 mg/l and found to be 2.3 fold high than the reference population (RP). *St. aegypti* WP of Borgong, Kusumtola and Serajuli displayed a RR_99_ of 2.5, 5.4 and 4.5 respectively suggesting high level of resistance to temephos. Results suggested that both *St. albopicta* and *St. aegypti* WP were fully resistant to DDT (mortality < 90%) in all the study locations. Both the species were completely susceptible to deltamethrin and malathion (corrected mortality > 98%), except for *St. albopicta* at Sotia which displayed low level of resistance to malathion (corrected mortality =95.4%). The estimated KDT values for both the species indicated high level of knock-down resistance to DDT and susceptibility to deltamethrin.

**Conclusion:**

WP of both the dengue vectors showed varied response to temephos, while resistant to DDT and completely susceptible to deltamethrin. Both the species were susceptible to malathion at majority of the testing sites. Current results strongly advocate that DDT is no longer effective against dengue vectors, while thorough monitoring of malathion susceptibility in geographical area at dengue risk is inexorable to ascertain whether or not the resistance to malathion is focal. Information generated herein may be useful in better planning and implementing in dengue control strategy using insecticides.

## Background

Dengue is the most important and rapidly expanding mosquito-borne viral infection, which is often inapparent but may develop into potentially lethal complications. The World Health Organisation (WHO) has estimated over 30-fold increase in global dengue incidences during the past 5 decades. About 50–100 million dengue cases are reported annually and almost half the world’s population is at risk of dengue infection [1]. Although dengue fever is a global concern now, at least 75% of the population exposed to dengue lives in the Asia-Pacific region. In India, dengue is spreading into the new areas and emerged as a major public health problem in the recent years. The effort to restrain dengue transmission primarily focuses on the vector control using insecticides and reducing mosquito breeding sites. However, the control efforts targeting efficient vectors *St. albopicta* and *St. aegypti* have failed to curb the increasing incidence of dengue epidemics and its invasion into the new geographical areas [2-4].

Dengue interventions using insecticides mainly target the immature stages of mosquitoes which breed in the artificial containers near the human houses and adult stages which actually transmit the dengue virus. The organophosphate (OP) compound temephos has been used to control the immature stage of dengue vectors for some time now [5,6] and probably resulted in the development of temephos resistance in many American [6] and Asian countries [7-9]. In India, although the dengue vector has been reported to be sensitive to temephos in many places [10,11], but as a consequence of widespread use the extent of temephos resistance may have been underestimated due to the lack of comprehensive data from different regions.

DDT, deltamethrin and malathion are commonly used insecticides to control the adult mosquito vectors, however resistance to these insecticides has been reported in many mosquito vectors in different countries [8,9,12-16]. Recent studies from endemic areas of India have reported the DDT resistance in dengue and other mosquito vectors, however there was decreased susceptibility to malathion, permethrin, deltamethrin, lambda-cyhalothrin and cyfluthrin in the majority of the tested mosquito vectors [11,14-16]. Not much data at regional level is available on the resistance status of dengue vectors in India, therefore evaluation of the efficacy of commonly used insecticides in different settings is inevitable under present circumstances.

The study area is endemic to malaria, however in recent years dengue cases have increased significantly [4,17]. Therefore the present study was conducted to determine the resistance level of field collected dengue vectors *St. albopicta* and *St. aegypti* in Sonitpur district of Assam to generate practical information which may be useful for taking up more effective vector control measures in future.

## Methods

### *Stegomyia* mosquito collections

**Figure 1:**
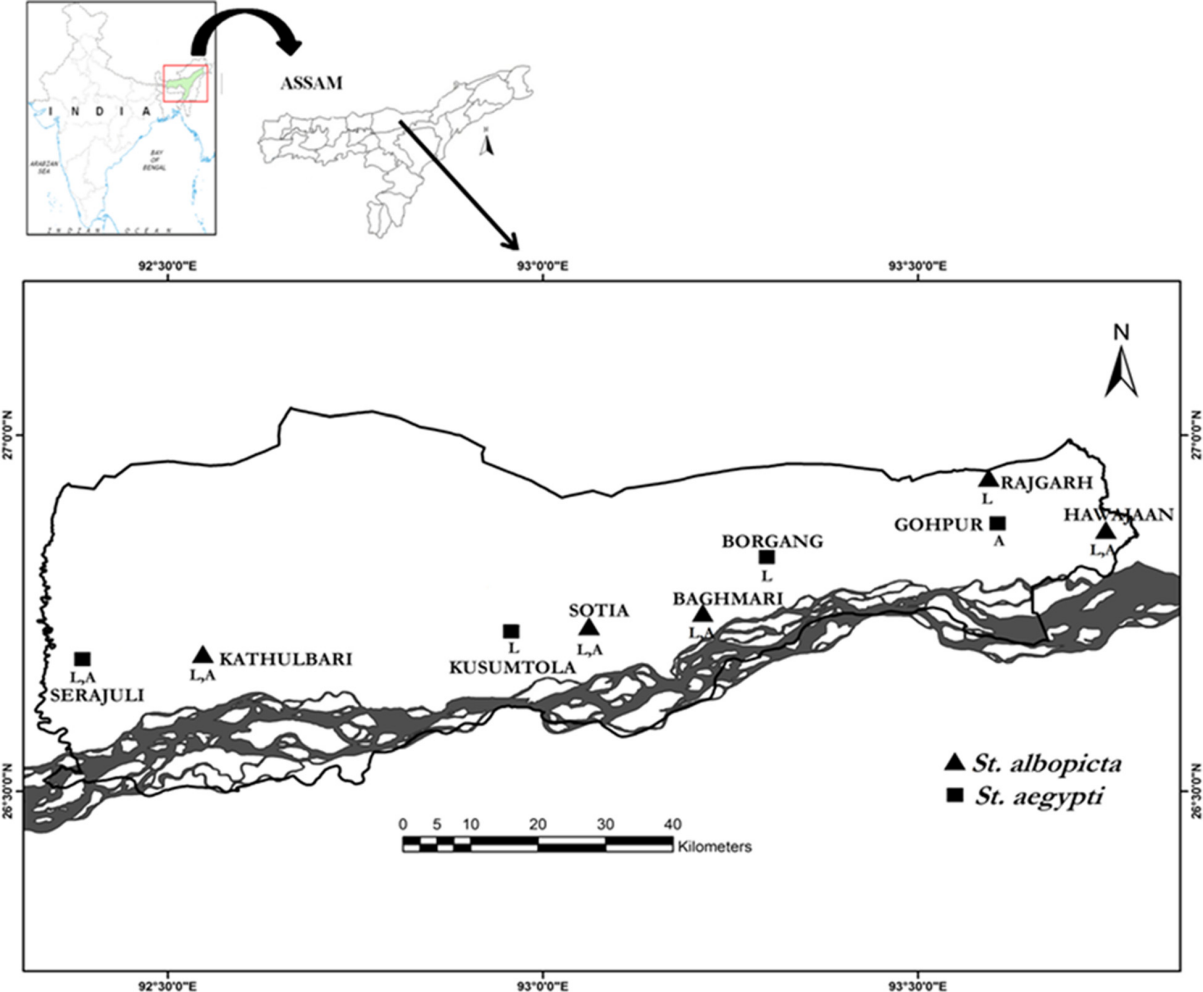
**GPS map of the study area depicting the** ***Stegomyia*** **mosquito sampling locations (L- larvae; A- adult).**

*St. albopicta* and *St. aegypti* mosquitoes (immature stages; F_0−_ generation) were sampled from nine localities in Sonitpur district of Assam, India (Figure 1) during March-October 2013. For each collection site, mosquitoes were collected from 6–8 breeding places in domestic, peri-domestic and natural environments. Mosquitoes were stored in plastic tubes containing water from the same breeding habitat and transferred to the laboratory. In laboratory, the WP of *Stegomyia* mosquitoes collected from different locations were reared separately to the adults and the F1-generation was used for larval and adult bioassays. Additionally, a *St. aegypti* population was also collected from Nameri National Park (GPS location: 27.0100° N, 92.7900° E), which is sylvan and hence considered as a susceptible reference population (RP) for wild collected *St. aegypti* populations (WP). *St. albopicta* mosquitoes are regularly maintained at the insectary of Defence Research Laboratory, Tezpur at controlled conditions (28°C ± 2°C; 80% ±10% RH) and were taken as RP for the *St. albopicta* WP mosquitoes.

### Larval susceptibility tests

Larval sensitivity to temephos (90.7% pure; Heranba Industries Ltd., Mumbai) was determined using standard WHO bioassay [18]. Temephos stock solution of 1 ppm and subsequent dilutions were prepared in 95% ethanol and stored at +4°C for use in the susceptibility tests. Susceptibility tests were conducted using 15–25 third instar larvaes (both WP and RP) in plastic disposable cups filled with required concentration of insecticide solution and millipore water (Milli-Q, MA, USA) at room temperature (28°C ± 2°C). Four different concentrations (0.001, 0.005, 0.01 and 0.05 ppm) were evaluated and each test was repeated at least three times [16]. The tests were accompanied by control tests to which only 95% ethanol in equal concentration was added into the water. Mortality was calculated after 24 hours of exposure.

### Adult insecticide susceptibility tests

Adult insecticide susceptibility bioassays were carried out using WHO test kits [19]. The insecticides impregnated papers used were obtained from Vector Control Research Unit (VCRU), Universiti Sains Malaysia, Malaysia. Bioassays were conducted using 4% DDT, 0.05% deltamethrin and 5% malathion. For DDT, the diagnostic concentration as per WHO criteria was used whereas for deltamethrin and malathion, the diagnostic concentrations from previous studies were used [11,16,20,21]. Unfed females (3–5 days old) were exposed to the insecticide impregnated papers in the batches of 10–15 for 60 minutes and cumulative knock-down was recorded after every 5 minutes. After 60 minutes, the survived mosquitoes were transferred to WHO holding tubes and fed on 5% sucrose solution for the next 24 hours. Mortality was recorded after 24 hours and graded for sensitivity status [22]. The control tests were performed using silicone oil, olive oil and risella oil pre-impregnated papers for deltamethrin, malathion and DDT respectively.

### Data analysis

Mortality was corrected using Schneider-Orelli's formula [23] and interpreted following WHO recommendation to determine the susceptibility status. Larval bioassay data were analyzed using Log dose probit (Ldp) Line computer programme according to Finney method [24]. Chi-square (χ^2^) test was used to estimate the goodness of fit, while liner regression (r^2^) was used to evaluate the data linearity. Lethal concentrations (LC_10_, LC_50_ and LC_99_) along with the slope were estimated at 95% confidence intervals (CI). The resistance ratios (RRs) (RR_50_ and RR_99_) were calculated by dividing the LC_50_ and LC_99_ values of WP with that of RP. A RR_99_ of < 2 corresponded to susceptible, whereas > 2 was considered corresponding to the resistance. In adult bioassays, knock-down time (KDT_10,_ KDT_50_ and KDT _99_) were determined using Ldp software, where overall significance of the multiple-tests was determined following Bonferroni procedure. The knock-down resistance ratios (KRRs) (KRR_50_ and KRR_99_) were calculated by dividing the KDT_50_ and KDT_99_ values of WP with that of RP. A KRR_99_ of < 2 implied susceptible, while > 2 was considered implying knock-down resistance in the WP *St. albopicta* and *St. aegypti* mosquitoes [16].

## Results

### *St. albopicta* and *St. aegypti* susceptibility to temephos

Temephos susceptibility to *St. albopicta* was determined at Hawajan, Baghmari, Sotia and Kathulbari areas which suggested that all the populations were completely susceptible except in Sotia where the LC_99_ value was 0.12 mg/l and RR_99_ was 2.3 (Table 1). The RR_50_ and RR_99_ values among the study populations ranged from 1.4- 1.5 and 0.9- 2.3 respectively. The LC values obtained for *St. albopicta* populations did not follow normal distribution for mortality to the log dose (χ^2^ ≥ 6.7; p ≤ 0.01). For *St. aegypti*, the populations of Rajgarh, Borgong, Kusumtola and Serajuli were tested for temephos sensitivity (Table 2). In Borgong, Kusumtola and Serajuli WP, the RR_99_ were 2.5, 5.4 and 4.5 respectively. The *St. aegypti* WP collected in Rajgarh was found to have incipient resistance as the RR_50_ and RR_99_ values were 1.9 each. The LC values obtained for *St. aegypti* larval bioassays in all the study populations did not exhibit linear probit between the log dose and mortality (χ^2^ ≥ 7.8; p ≤ 0.02).

**Table 1:** **Temephos sensitivity of** ***St. albopicta*** **larvae and resistance ratio (RR) in the study populations**

**Values**	**Hawajan (N = 675)**	**Baghmari (N = 675)**	**Sotia (N = 675)**	**Kathulbari (N = 675)**	**RS (N = 350)**
LC_10_	0.0004	0.0004	0.0004	0.0006	0.0004
LC_50_	0.0028	0.0029	0.0029	0.0027	0.002
LC_99_	0.079	0.092	0.12	0.044	0.052
χ^2^ (p)	10.8 (0.004)	11.7 (0.003)	6.7 (0.01)	13.7 (0.001)	1.3 (0.6)
Slope	1.60 ± 0.1	1.55 ± 0.1	1.44 ± 0.2	1.89 ± 0.2	1.64 ± 0.2
r	0.98	0.97	0.96	0.97	0.99
g	0.68	0.74	13.6	0.89	0.30
RR_50_ (95% CI)/ RR_99_ (95% CI)	1.4 (1.2-1.7)/ 1.5 (1.3-1.8)	1.5 (1.2-1.7)/ 1.8 (1.5-2.1)	1.5 (1.2-1.7)/ 2.3 (2.0-2.6)	1.4 (1.1-1.6)/ 0.9 (0.7-1.1)	-

N- number; LC10/50/99- lethal concentration 10%/50%/99%.

**Table 2:** **Temephos sensitivity of** ***St. aegypti*** **larvae and resistance ratio (RR) in the study populations**

**Values**	**Rajgarh (N = 675)**	**Borgong (N = 675)**	**Kusumtola (N = 675)**	**Serajuli (N = 675)**	**RS (N = 250)**
LC_10_	0.0005	0.0005	0.0004	0.0005	0.0003
LC_50_	0.0031	0.0035	0.0037	0.0041	0.0016
LC_99_	0.086	0.11	0.24	0.2	0.044
χ^2^ (p)	11.1 (0.004)	12.6 (0.002)	8.7 (0.01)	7.8 (0.02)	0.4 (0.5)
Slope	1.60 ± 0.1	1.54 ± 0.1	1.28 ± 0.1	1.38 ± 0.1	1.60 ± 0.3
r	0.98	0.97	0.98	0.98	0.99
g	0.67	0.77	0.73	0.60	0.10
RR_50_ (95% CI)/ RR_99_ (95% CI)	1.9 (1.6-2.2)/ 1.9 (1.6-2.1)	2.2 (1.9-2.4)/ 2.5 (2.2-2.8)	2.3 (2.0-2.6)/ 5.4 (4.9-5.9)	2.6 (2.3-2.9)/ 4.5 (4.1-5.0)	-

N- number; LC10/50/99- lethal concentration 10%/50%/99%.

### Insecticides toxicity to field populations of adult *St. albopicta* and *St. aegypti*

Adult insecticide susceptibility bioassay for *St. albopicta* were performed in WP of Hawajan, Sotia and Kathulbari, while for *St. aegypti*, the bioassays were carried out for WP of Gohpur, Kusumtola and Serajuli. The results of adult susceptibility bio-assay for *St. albopicta* and *St. aegypti* have been shown in Tables 3 and 4 respectively. After 24 hours post exposure, the percent corrected mortality obtained for DDT ranged from 45.2 to 59.4 among the *St. albopicta* populations, while 65.0 to 70.5 among the *St. aegypti* populations. However the corrected mortality obtained in RP of *St. albopicta* was 80.0-85.0%, where in *St aegypti* varied from 84.5 to 88.0 percent against DDT. Results suggested that both *St. albopicta* and *St. aegypti* WP were fully resistant to DDT in all the study locations. Both the species were completely susceptible to deltamethrin and malathion (corrected mortality > 98%), except for *St. albopicta* at Sotia which showed low level of resistance to malation (corrected mortality = 95.4%). The mortality obtained in *St. albopicta* RP was 100% for deltamethrin and 95-100% for malathion. Similarly for *St. aegypti* RP, the percent corrected mortality against both deltamethrin and malathion was 100%. The KDT values determined for DDT and deltamethrin for all the study WP and malathion for *St. aegypti* Kusumtola WP displayed a linear probit for knock-down rates with time (Tables 3 and 4). The estimated KDT of DDT for *St. albopicta* indicated that KDT values (both KDT_50_ and KDT_99_) for WP were > 2 folds (2.1- 4.7) higher to those of RP except for KDT_50_ in Hawajan WP (1.1 folds). In case of *St. aegypti* also there was > 2 fold rise in the KDT_50_ (2.1- 2.4) and KDT_99_ (2.1- 3.3) among the test populations. The results suggest that high level of DDT knock-down resistance was present among both the *Stegomyia* species. For deltamethrin, the KRR_50_ and KRR_99_ values were below 2 in all the test populations of both the species which indicates the knock-down sensitivity among the test WP. For malathion, both KRR_50_ and KRR_99_ for *St. albopicta* ranged from 1.7 to 1.9 (for KRR_50_) and 1.9 to 2.1 (for KRR_99_) in the test populations. However for *St. aegypti* WP, the KRR_50_ (2.1- 2.9) and KRR_99_ (2.6- 3.7) values were > 2 folds higher in all the test populations as compared to the RP. *St. aegypti* was confirmed knock-down resistant to malathion whereas *St. albopicta* may have developed low level of knock-down resistance in Sotia WP.

**Table 3:** **Mortality and knock-down in** ***St. albopicta*** **(WP) against different insecticides**

	**Insecticide (N)**	**CM (%)**	**KDT**_10_ **(95%** **CI)**	**KDT**_50_ **(95%** **CI)**	**KDT**_99_ **(95%** **CI)**	**χ** ^**2**^ **(p)**	**Slope**	**KRR**_50_ **/ KRR**_99_	**95%** **CI for KRR**_50_ **/KRR**_99_
**Hawajan**	DDT (106)	59.4	25.6 (22.3-28.4)	71.1 (62.8-84.7)	452.0 (296.3-853.5)	6.0 (0.7)	2.9 ± 0.2	1.1/2.6	0.9-1.3/2.3-2.9
	DM (113)	100.0	3.2 (2.4-3.9)	9.6 (8.5-10.7)	72.5 (58.3-96.4)	6.8 (0.2)	2.7 ± 0.2	1.7/1.6	1.4-1.9/1.4-1.9
	MA (107)	99.1	25.0 (18.4-28.4)	83.0 (73.9-127.7)	731.7 (601.3-3009.4)	35.1 (<0.0001)	2.5 ± 0.2	1.7/1.9	1.5-1.9/1.6-2.2
**Sotia**	DDT (163)	50.8	26.7 (21.9-30.5)	92.1 (74.2-132.9)	872.8 (435.9-3001.7)	6.8 (0.7)	2.4 ± 0.3	2.1/4.7	1.8-2.4/4.3-5.1
	DM (182)	98.8	4.0 (3.1-4.8)	9.3 (8.3-10.2)	43.1 (36.1-54.2)	5.4 (0.3)	3.5 ± 0.3	1.1/1.2	0.9-1.3/1.0-1.5
	MA (105)	95.4	27.6 (15.2-30.9)	77.6 (74.6-186.7)	508.8 (495.9-1203.2)	50.3 (<0.0001)	2.8 ± 0.4	1.8/2.1	1.6-2.1/1.8-2.4
**Kathulbari**	DDT (126)	45.2	26.2 (23.2-28.8)	84.8 (73.5-103.7)	712.8 (45.6-1418.4)	14.2 (0.1)	2.5 ± 0.2	2.1/3.3	1.8-2.4/2.9-3.7
	DM (112)	100.0	3.3 (2.5-4.1)	8.0 (7.0-8.9)	39.3 (32.9-49.5)	7.0 (0.2)	3.4 ± 0.3	0.9/1.0	0.7-1.1/0.8-1.2
	MA (113)	99.1	21.2 (15.4-24.2)	71.7 (63.1-101.8)	654.1 (532.2-2116.5)	36.7 (<0.0001)	2.4 ± 0.2	1.9/1.9	1.6-2.2/1.7-2.2

WP- wild population; N- number; CM- corrected mortality; CI- confidence interval; DM- deltamethrin; MA- malathion.

**Table 4:** **Mortality and knock-down in** ***St. aegypti*** **(WP) against different insecticides**

	**Insecticide (N)**	**CM (%)**	**KDT**_10_ **(95%** **CI)**	**KDT**_50_ **(95%** **CI)**	**KDT**_99_ **(95** **%** **CI)**	**χ** ^**2**^ **(p)**	**Slope**	**KRR**_50_ **/ KRR**_99_	**95%** **CI for KRR**_50_ **/KRR**_99_
**Gohpur**	DDT (116)	70.5	23.0 (19.1-26.1)	65.5 (57.5-79.6)	441.1 (276.5-929.0)	3.2 (1.0)	2.8 ± 0.3	2.4/2.3	2.1-2.7/1.9-2.7
	DM (177)	100.0	3.2 (2.4-4.0)	7.0 (6.1-7.8)	28.8 (23.2-39.6)	2.1 (0.3)	3.8 ± 0.4	1.3/1.2	1.1-1.5/1.0-1.4
	MA (100)	100.0	19.0 (12.5-22.3)	64.5 (55.1-95.0)	592.1 (27.8-2510.7)	25.4 (0.005)	2.4 ± 0.3	2.9/2.9	2.6-3.2/2.6-3.3
**Kusumtola**	DDT (142)	69.0	23.7 (17.9-28.0)	83.2 (66.2-126.1)	813.0 (78.4-3640.0)	5.7 (0.8)	2.3 ± 0.4	2.1/2.1	1.8-2.4/1.8-2.4
	DM (150)	98.0	3.4 (1.6-4.7)	10.1 (8.2-12.1)	53.5 (39.5-107.5)	3.1 (0.08)	2.7 ± 0.6	1.6/1.7	1.4-1.9/1.5-1.9
	MA (150)	100.0	21.9 (17.1-25.6)	76.8 (63.5-104.4)	751.2 (90.9-2338.4)	8.1 (0.6)	2.3 ± 0.3	2.5/2.6	2.2-2.9/2.3-2.9
**Serajuli**	DDT (92)	65.0	22.5 (17.1-26.6)	99.5 (76.6-158.9)	1483.5 (618.5-7662.7)	6.8 (0.7)	2.0 ± 0.3	2.2/3.3	1.9-2.5/2.9-3.7
	DM (116)	100.0	3.8 (1.1-5.4)	11.7 (10.1-14.3)	39.6 (34.9-46.6)	9.1 (0.2)	3.2 ± 0.3	1.3/1.4	1.1-1.5/1.2-1.7
	MA (100)	98.3	14.1 (6.5-16.1)	65.6 (55.8-125.5)	1063.8 (1033.6-13912.4)	37.9 (0.0)	1.9 ± 0.2	2.1/3.7	1.8-2.4/3.3-4.1

WP- wild population; N- number; CM- corrected mortality; CI- confidence interval; DM- deltamethrin; MA- malathion.

## Discussion

Northeastern region of India is geographically isolated from the mainland of India and has been an important centre of mosquito-borne diseases in India. Mosquito vector control using insecticides is the primary element which argues that determination of insecticide resistance status of mosquitoes is inevitable. Current study has investigated the insecticide resistance status of *St. albopicta* and *St. aegypti* and determined the knock-down resistance in some sentinel locations in Assam state of northeastern India.

Present study found that the majority of the field populations of *St. albopicta* were susceptible to temephos (RR_99_ < 2), however in Sotia, the RR_99_ of 2.3 suggested that an incipient level of temephos resistance was present in the test population as compared to the RP. The LC_50_ and LC_99_ values were quite high for *St. aegypti* field populations. The LC_50_ ranged from 0.0031 in Rajgarh WP to 0.0041 in Serajuli WP, while the LC_99_ ranged from 0.086 in Rajgarh to 0.24 in Kusumtola WP respectively. Both RR_50_ and RR_99_ values were found to be above 2 in the *St. aegypti* field populations of Borgang, Kusumtola and Serajuli which strongly evidences the development of resistance in these populations. The RR values in *St. aegypti* WP in Rajgarh were 1.9 each and suggested that a high level of physiological resistance may develop shortly due to continuous selection pressure.

In the present study although the LC_99_ value obtained for both the mosquito species were below the recommended dose of application of temephos as larvicide [4,25], but are above the LC_99_ values obtained for the RP. The principal intervention to control dengue vector in India involves the application of temephos formulation not exceeding the concentration of 1 mg/l to the domestic and peridomestic water containers, but it is not considered appropriate to set a formal guideline value for the insecticides as vector control agents in water [5]. Therefore determining dose for the control of vector mosquitoes and their level of sensitivity to larvicide at local level is important.

Resistance to temephos has been reported in many Southeast Asian countries [7-9]. However, the present results have suggested that *St. albopicta* is susceptible to temephos but the mortality data did not follow linear trend with the dose. A recent study has suggested that *St. albopicta* larvae were susceptible to temephos in northeast India and the data obtained followed linear trend of mortality with dose [16]. Similarly, another study in the urban setting of Assam, India suggested that both *St. aegypti* and *St. albopicta* were completely sensitive to temephos and the LC_95_ concentrations were about 66 folds lower for *St. aegypti* and about 345 folds lower for *St. albopicta* as compared to the standard diagnostic concentration [4].

A high level of physiological resistance to insecticides could develop under the influence of selection pressure. A previous study has shown that the offspring of the survivors of selection pressure exhibited significantly reduced mortality and displayed about 298 folds increase in the LC_50_ values as compared to the F0 colony [26]. The RR values for the most efficient dengue vector *St. aegypti* in majority of populations rose above 2 probably due to the selection of insecticide. The results suggest that *St. aegypti* WP were more resistant than the *St. albopicta* WP as the LC values and RR were higher for *St. aegypti* populations. *St. aegypti* might be under the influence of insecticide selection because is prefers to rest indoors and probably experiences more exposure to the household insecticides as compared to *St. albopicta* which opts to resting outdoors [7].

DDT use is widespread for controlling malaria vector mosquitoes and other vector-borne diseases in the region, whereas synthetic pyrethroid are used in long lasting insecticidal bed nets (LLIN) [14,15]. Adult susceptibility bioassay results suggest that field populations of known dengue vectors *St. albopicta* and *St. aegypti* displayed a high level of phenotypic resistance to DDT. The corrected mortality obtained for DDT in different populations of *St. albopicta* ranged from 45.2 to 59.4, while for *St. aegypti* ranged from 65 to 70.5. Interestingly the corrected mortality was higher in *St. aegypti* populations as compared to *St. albopicta* suggesting that *St. aegypti* were comparatively more sensitive to DDT. The RP of both the species used in the present study have shown mortality ranging between 80.0-88.0 percent for DDT, which indicated that a considerable level of DDT resistance has developed even in the laboratory reared population. The KDT_99_ value for *St. albopicta* ranged from 452.0 minutes in Hawajan to 872.8 minutes in Sotia, while for *St. aegypti* the KDT_99_ values ranged from 441.1 in Gohpur to 1483.5 in Serajuli. The KDT values obtained for DDT in the current study have suggested that both the tested species populations have developed potential knock-down resistance to DDT as compared to the RP. DDT resistance among the different species of vector mosquitoes has been well reported during recent years [4,14-16,26]. Previous studies have suggested that continuous and indiscreet use of DDT in vector-borne disease control intervention has led to the development and spatial spread of resistance among many efficient vector mosquitoes [4,11,14-16,26]. Our results are in line with the previous study which demonstrated that potential dengue vector *St. albopicta* was knock-down resistant to DDT, while sensitivity to malathion and deltamethrin was potentially reduced [16]. Resistance to DDT in *Stegomyia* mosquitoes has been associated with the mutation in kdr gene of voltage-gated sodium channel, however increased enzyme activity of detoxifying enzymes such as glutathione S-transferases and mixed function oxidases can also play a key role in DDT resistance [27-29]. Although many studies have recorded DDT resistance in *Stegomyia* mosquito in India and neighbouring countries the underlying mechanisms largely remain unclear [4,11,16,21].

All the mosquito populations were completely susceptible to deltamethrin and the corrected mortality ranged from 98 to 100 percent. Both KRR_50_ and KRR_99_ values were below 2 for both the species in all the populations which indicated the knock-down susceptibility of mosquitoes. The probit model suggested that the deltamethrin KDT values obtained for each population displayed a linear relationship. A varied level of response to deltamethrin and permethrin was observed in a recent study, where *St. aegypti* were resistant to deltamethrin as well as permethrin, but *St. albopicta* were susceptible to deltamethrin [4]. Recently, synthetic pyrethroid have been used tremendously in mosquito control which has reduced the sensitivity of mosquito to pyrethroids in many regions [4,8,9,28,29] under the influence of multiple mechanisms [30], however the current results suggest that synthetic pyrethroids are equally effective against the dengue vectors. The results further emphasize that the resistance to DDT has not conferred any cross resistance to deltamethrin. Malathion is used widely in the control of mosquito vectors during epidemics in most parts of India, however the resistance to malathion in dengue vectors has not been reported in the northeastern region of India [4,16]. The test populations of both the species were sensitive to malathion as the corrected mortality was above 98 percent, however *St. albopicta* WP of Sotia exhibited 95.4 percent mortality which suggests the developed lower level of resistance to malathion. The KRR_99_ value of Sotia WP was 2.1, which further confirms the incipient level of knock-down resistance. Interestingly the KRR values for *St. aegypti* were well above 2 among all the test populations which shows evidence that there has been development and spread of knock-down resistance against malathion in the region. Recent studies have reported that dengue vectors are fully susceptible however the KDT_50_ in *St. albopicta* was found to be 3.2 folds higher than the reference [4,16]. Considering the fact that malathion is routinely used in the anti-mosquito fogging operations during the peak epidemic periods of Japanese encephalitis and dengue in northeastern states, the dengue vectors are at continuous selection which might reduce sensitivity to malathion.

The purpose of this study was to evaluate the insecticide resistance status of larval and adult stages of *St. albopicta* and *St. aegypti* populations in different places of Assam to verify the expected success in dengue vector control programmes using insecticides. Insecticide resistance is vital, which can potentially compromise the mosquito vector control efforts and affect the efficiency of insecticides under field conditions. In India, DDT use is widespread in vector control intervention but its role against *Stegomyia* mosquitoes appears to be limited since it is largely used in indoor sprays [14,15,25]. However it is possible that excessive and routine use of DDT exerted indirect selection pressure on both the *Stegomyia* species. Previous studies have suggested that long-term use of DDT in different vector control programmes has led to DDT selection in *Stegomyia* and other mosquito species [12,30,31]. Therefore, monitoring and detecting resistance at early stage may improve vector control interventions by pre-emptively triggering the evidence based implementation of alternative control strategies for achieving promising results [32]. Since dengue vector is expanding its spread rapidly in northeastern states, the spatial data on the effectiveness of insecticides used in control measures is crucial.

## Conclusion

Current research has generated sufficient data on the susceptibility status of two dengue vector species for selecting insecticide in the dengue control operations. Both *St. albopicta* and *St. aegypti* populations were completely resistant to DDT, but completely susceptible to deltamethrin. There has been a decrease in the susceptibility to malathion in adult stages and susceptibility to temephos in larval stages in some of the study populations. We recommend the regular monitoring of insecticide resistance in a large geographical area to pin-point the level of resistance in different areas to warrant the use of insecticides to which even a minimum level of resistance is observed.
